# Supplementary material for: Analysis of Selected Nutritional Parameters in Patients with HPV-Related and Non-HPV-Related Oropharyngeal Cancer before and after Radiotherapy Alone or Combined with Chemotherapy
Source: Cancers (Basel). 2022 May 9;14(9):2335. doi: 10.3390/cancers14092335 (PMC9101210; doi:10.3390/cancers14092335)
Supplement: Supplementary file 1 [file cancers-14-02335-s001.zip › cancers-1666344-supplementary.pdf]

Analysis of Selected Nutritional Parameters in Patients with HPV-Related and HPV- not Related Oropharyngeal Cancer Before and After Radiotherapy Alone or Combined with Chemotherapy

Adam Brewczyński[1], Beata Jabłońska[2*], Agnieszka Maria Mazurek[3], Jolanta Mrochem-Kwarciak[4], Sławomir Mrowiec[2], Mirosław Śnietura[5], Marek Kentnowski[1], Anna Kotylak[1], Zofia Kołosza[6], Krzysztof Składowski[1] and Tomasz Rutkowski[1]

Supplementary materials:

Table S1. Laboratory results in patients before and after treatment.

Table S2. Weight, BMI and laboratory results: differences before and after treatment in HPV- and HPV+ groups undergoing RT and CRT separately (comparison of values before and after RT/CRT within individual HPV- and HPV+ subgroups + separately).

Table S3. Weight, BMI and laboratory results before and after treatment in HPV- and HPV+ groups undergoing RT and CRT separately (comparison of difference values between two subgroups according to HPV status).

Table S4. Comparison of selected clinicopathological factors between groups with low and high prognostic nutritional index (PNI).

Table S5. Overall survival (OS) in patients: univariate and multivariate analysis for pre-treatment parameters.

Table S6. Disease-free survival (DFS) in patients: univariate and multivariate analysis for pre-treatment parameters.

Figure S1. Overall and disease-free survival in HPV- and HPV+ patients depending on NRS 2002 (NRS 2002<3 vs. NRS 2002≥3).
Figure S2. Overall and disease-free survival in HPV- and HPV+ patients depending on NRS 2002 (weight loss<5% vs. weight loss>5%).

Table S1. Laboratory results in patients before and after treatment.

	All	HPV(-)	HPV(+)	p	*p* HB
CRP 0 [g/l]	5.15±7.07 (0.16-36.90)	6.79±8.39 (0.16-36.90)	3.40±4.80 (0.17-27.20)	0.008	0.088
CRP 1 [g/l]	26.60±35.93 (0.17-197.00)	30.96±44.43 (0.20-197.00)	22.00±23.50 (0.17-87.20)	0.179	1.000
Albumin 0 [g/l]	41.67±3.68 (33.00-50.00)	41.58±3.65 (33.00-49.00)	41.77±3.74 (32.00-50.00)	0.778	1.000
Albumin 1 [g/l]	36.95±3.92 (30.00-49.00)	37.05±4.11 (30.00-47.00)	36.84±3.75 (30.00-49.00)	0.778	1.000
Prealbumin 0 [g/l]	0.28±0.08 (0.13-0.52)	0.26±0.09 (0.13-0.52)	0.29±0.07 (0.18-0.45)	0.092	0.920
Prealbumin 1 [g/l]	0.20±0.08 (0.08-0.49)	0.19±0.07 (0.08-0.35)	0.21±0.09 (0.09-0.49)	0.223	1.000
Haemoglobin 0 [g/dl]	13.97± 1.50 (10.60-17.40)	14.05±1.51 (12.85-15.35)	13.88±1.51 (10.80-16.90)	0.530	1.000
Haemoglobin 0 [g/dl]	12.70± 1.50 (9.40-16.30)	12.29±1.46 (9.40-16.30)	12.03±1.54 (9.50-15.80)	0.331	1.000

TLC 0 [/mm^3]	1.90±0.72 (0.57-4.84)	1.91±0.71 (0.65-3.94)	1.89±0.73 (0.57-4.54)	0.842	0.842
TLC 1 [/mm^3]	0.62±0.37 (0.11-2.84)	0.71±0.44 (0.11-2.84)	0.52±0.24 (0.17-1.34)	0.004	0.048
PNI 0	41.68±3.68 (32.00-50.00)	41.59±3.65 (33.01-49.01)	41.78±3.74 (32.00-50.00)	0.778	1.000
PNI 1	36.95±3.92 (30.00-49.00)	37.05±4.11 (30.00-47.00)	36.85±3.75 (30.00-49.00)	0.777	1.000

Values are presented as means and standard deviations.

0, before treatment, 1 after treatment; CRP, C-reactive protein; TLC, total lymphocyte count; PNI, prognostic nutritional index.

H-B Holm-Bonferroni correction for multiple testing

Table S2. Weight, BMI and laboratory results: differences before and after treatment in HPV- and HPV+ groups undergoing RT and CRT separately (comparison of values before and after RT/CRT within individual HPV- and HPV+ subgroups + separately).

	CRT		*p*	*p* H-B	RT		*p*	*p* H-B
	HPV(-)	HPV(+)			HPV(-)	HPV(+)		
Weight 0-1 [kg]	4.11±3.75	5.76±4.27	0.142	1.000	4.89±3.68	4.83±4.21	1.000	1.000
Weight [%]	5.62±5.24	7.32±5.24	0.221	1.000	5.73±3.74	5.13±4,55	1.000	1.000
BMI 0-1 [kg/m^2]	1.44±1.34	1.99±1.40	0.127	1.000	1.84±1.37	1.81±1.70	1.000	1.000
CRP 0-1 [g/l]	-19.70±37.60	-17.92±22.07	0.623	1.000	-32.39±55.67	-24.79±32.97	0.803	1.000
Albumin 0-1 [g/l]	4.89±4.37	5.00±5.05	0.774	1.000	3.75±4.33	5.14±4.53	0.221	1.000
Prealbumin 0-1 [g/l]	0.059±0.101	0.088±0.098	0.237	1.000	0.079±0.087	0.002±0.116	0.431	1.000
Haemoglobin 0-1 [g/dl]	2.10±1.54	2.04±1.55	0.943	1.000	1.10±1.05	0.44±1.13	0.249	1.000
TLC 0-1 [/mm^3]	1.22±0.77	1.36±0.70	0.438	1.000	1.15±0.63	1.46±0.69	0.351	1.000
PNI 0-1	4.90±1.37	5.01±5.06	0.739	1.000	3.76±4.33	5.15±4.53	0.213	1.000

Table S3. Weight, BMI and laboratory results before and after treatment in HPV- and HPV+ groups undergoing RT and CRT separately (comparison of difference values between two subgroups according to HPV status).

	CRT		*p*	*p* H-B	RT		*p*	*p* H-B
	HPV(-)	HPV(+)			HPV(-)	HPV(+)		

Weight 0 [kg]	72.60±14.13	78.58±14.92	0.091	1.000	71.99±17.04	85.53±11.43	0.050	1.000
Weight 1[kg]	68.96±13.24	72.10±14.71	0.560	1.000	75.08±13.44	86.73±9.72	0.276	1.000
BMI 0 [kg/m^2]	25.11±4.00	27.78±4.31	0.007	0.241	26.92±5.69	30.52±3.58	0.103	1.000
BMI 1 [kg/m^2]	23.56±3.21	25.40±4.10	0.090	1.000	27.91±4.53	30.91±3.47	0.350	1.000
NRS 2002 0	0.36±0.84	0.20±0.40	0.883	1.000	0.50±0.93	0.86±1.46	0.615	1.000
NRS 2002 1	3.06±1.15	3.40±1.12	0.217	1.000	2.73±0.90	3.33±0.58	0.314	1.000
CRP 0 [g/l]	7.18±9.76	2.94±3.77	0.016	0.546	6.02±4.84	6.81±9.24	0.730	1.000
CRP 1 [g/l]	27.13±35.78	20.66±22.49	0.770	1.000	38.63±58.38	31.59±30.05	0.978	1.000
Albumin 0 [g/l]	42.05±3.91	41.92±3.93	0.949	1.000	40.67±2.96	40.71±1.80	0.957	1.000
Albumin 1 [g/l]	37.23±4.16	37.02±3.77	0.899	1.000	36.71±4.10	35.57±3.60	0.454	1.000
Prealbumin 0 [g/l]	0.277±0.079	0.301±0.070	0.117	1.000	0.230±0.098	0.213±0.029	0.861	1.000
Prealbumin 1 [g/l]	0.213±0.072	0.217±0.081	0.965	1.000	0.150±0.048	0.200±0.120	0.536	1.000
Haemoglobin 0 [g/dl]	14.08±1.50	13.85±1.56	0.466	1.000	14.00±1.54	14.09±1.10	0.887	1.000
Haemoglobin 1 [g/dl]	11.98±1.40	11.82±1.41	0.450	1.000	12.87±1.41	13.64±1.65	0.249	1.000
TLC 0 [/mm^3]	1.91±0.78	1.89±0.75	0.840	1.000	1.91±0.56	1.85±0.57	0.723	1.000
TLC 1 [/mm^3]	0.69±0.47	0.53±0.25	0.084	1.000	0.74±0.38	0.40±0.16	0.006	0.234
PNI 0	42.06±3.91	41.93±3.93	0.936	1.000	40.68±2.96	40.72±1.80	1.000	1.000
PNI 1	37.23±4.16	37.02±3.77	0.820	1.000	36.72±4.10	35.57±3.60	0.265	1.000

Table S4. Comparison of selected clinicopathological factors between groups with low and high prognostic nutritional index (PNI).

	All				HPV(-)				HPV(+)			
	**PNI<41.68**	**PNI≥41.68**	**p**	***p* HB**	**PNI<41.59**	**PNI≥41.59**	**p**	***p* HB**	**PNI<41.78**	**PNI≥41.78**	**p**	***p* HB**
Age [years]	62.8	58.5	0.004	0.076	62.5	59.2	0.031	1.00	57.7	63.2	0.004	0.148
Gender			0.235	1.00			0.764	1.00			0.184	1.00
M	38 (64.4%)	45 (75.0%)			24 (75.0%)	24 (80.0%)			14 (51.9%)	21 (70.0%)		
F	21 (35.6%)	15 (25.0%)			8 (25.0%)	6 (20.0%)			13 (48.1%)	9 (30.0%)		

General location			0.004	0.072			0.033	1.00			0.093	1.00
Tonsil	36 (61.0%)	51 (85.0%)			17 (53.1%)	24 (80.0%)			19 (70.4%)	27 (90.0%)		
Others	23 (39.0%)	9 (15.0%)			15 (46.9%)	6 (20.0%)			8 (29.6%)	3 (10.0%)		
Detailed location			0.005	0.085			0.031	1.00			0.120	1.00
1. tonsil	36 (61.0%)	51 (85.0%)			17 (53.1%)	24 (80.0%)			19 (70.4%)	27 (90.0%)		
2. palate	8 (13.6%)	1 (1.7%)			8 (25.0%)	1 (3.3%)						
3. root of the tongue	12 (20.3%)	8 (13.3%)			7 (21.9%)	5 (16.7%)			5 (18.5%)	3 (10.0%)		
4. other oropharynx	3 (5.1%)	0 (0%)			0	0			3 (11.1%)	0		
Histological grading			0.346	1.00			0.515	1.00			0.367	1.00
G1	3 (8.1%)	4 (10.8%)			3 (15.0%)	3 (14.3%)			0	1 (6.3%)		
G2	27 (73.0%)	21 (56.8%)			15 (75.0%)	13 (61.9%)			12 (70.6%)	8 (50.0%)		
G3	7 (18.9%)	12 (32.4%)			2 (10.0%)	5 (23.8%)			5 (29.4%)	7 (43.8%)		
			0.287	1.00			0.410	1.00			0.481	1.00
G1-2	30 (81.1%)	25 (67.6%)			18 (90.0%)	16 (76.2%)			12 (70.6%)	9 (56.3%)		
G3	7 (18.9%)	12 (32.4%)			2 (10.0%)	5 (23.8%)			5 (29.4%)	7 (43.8%)		
Tumor depth (T)			0.257	1.00			0.107	1.00			0.733	1.00
T1	5 (8.5%)	6 (10.0%)			3 (9.4%)	3 (10.0%)			2 (7.4%)	3 (10.0%)		
T2	19 (32.2%)	21 (35.0%)			13 (40.6%)	11 (36.7%)			6 (22.2%)	10 (33.3%)		
T3	18 (30.5%)	25 (41.7%)			7 (21.9%)	14 (46.7%)			11 (40.7%)	11 (36.7%)		
T4	16 (27.1%)	8 (13.3%)			8 (25.0%)	2 (6.7%)			8 (29.6%)	6 (20.0%)		
Tx	1 (1.7%)	0			1 (3.1%)	0						
			0.714	1.00			0.805	1.00			0.410	1.00
T1-2	24 (40.7%)	27 (45.0%)			16 (50.0%)	14 (46.7%)			8 (29.6%)	13 (43.3%)		
T3-4	34 (59.3%)	33 (55.0%)			16 (50.0%)	16 (53.3%)			19 (70.4%)	17 (56.7%)		
Lymph node metastasis (N)			0.366	1.00			0.771	1.00			0.476	1.00
N0	13 (22.0%)	10 (16.7%)			10 (31.3%)	7 (23.3%)			3 (11.1%)	3 (10.0%)		
N1	8 (13.6%)	16 (26.7%)			6 (18.8%)	9 (30.0%)			2 (7.4%)	7 (23.3%)		
N2	29 (49.2%)	26 (43.3%)			12 (37.5%)	10 (33.3%)			17 (63.0%)	16 (53.3%)		
N3	8 (13.6%)	8 (13.3%)			4 (12.5%)	4 (13.3%)			4 (14.8%)	4 (13.3%)		
Nx	1 (1.7%)	0							1 (3.7%)	0		
			0.455	1.00			0.805	1.00			0.241	1.00
N0-1	21 (35.6%)	26 (43.3%)			16 (50.0%)	16 (53.3%)			5 (18.5%)	10 (33.3%)		
N2-3	38 (64.4%)	34 (56.7%)			16 (50.0%)	14 (46.7%)			22 (81.5%)	20 (66.7%)		
Treatment duration [days]	46.1	46.3	0.233	1.00	43.3	44.9	0.292	1.00	47.7	49.4	0.233	1.00

General treatment regimen			0.394	1.00			0.598	1.00			0.697	1.00
RT	16 (27.1%)	12 (20.0%)			12 (37.5%)	9 (30.0%)			4 (14.8%)	3 (10.0%)		
CRT	43 (72.9%)	48 (80.0%)			20	21			23 (85.2%)	27 (90.0%)		
Weight loss			0.282	1.00			0.751	1.00			0.260	1.00
1. Stable												
2. Weight loss ≤ 5%	21 (50.0%)	17 (37.0%)			10 (50.0%)	8 (42.1%)			11 (50.0%)	9 (33.3%)		
3. Weight loss > 5%	21 (50.0%)	29 (63.0%)			10 (50.0%)	11 (57.9%)			11 (50.0%)	18 (66.7%)		
Initial BMI [kg/m^2]	27.0	26.8	0.922	1.00	25.5	26.0	0.816	1.00	27.6	28.8	0.922	1.00
BMI groups			0.679	1.00			1.00	1.00			0.474	1.00
1. <18.5	3 (5.1%)	2 (3.3%)			2 (6.3%)	2 (6.7%)			1 (3.7%)	0		
2. ≥18.5	56 (94.9%)	58 (96.7%)			30 (93.8%)	28 (93.3%)			26 (96.3%)	30 (100%)		
NRS 2002 (mean value)												
NRS 2002			0.547	1.00			0.701	1.00			0.145	1.00
0	43 (72.9%)	47 (79.7%)			25 (78.1%)	22 (73.3%)			18 (66.7%)	25 (86.2%)		
1.	13 (22.0%)	7 (11.9%)			5 (15.6%)	3 (10.0%)			8 (29.6%)	4 (13.8%)		
2.	1 (1.7%)	3 (5.1%)			1 (3.1%)	3 (10.0%)						
3.	1 (1.7%)	1 (1.7%)			1 (3.1%)	1 (3.3%)						
4.	1 (1.7%)	1 (1.7%)			0	1 (3.3%)			1 (3.7%)	0		
5.												
NRS 2002 (division into groups)			1.00	1.00			0.607	1.00			0.482	1.00
1. <3	57 (96.6%)	57 (96.6%)			31 (96.9%)	28 (93.3%)			26 (96.3%)	29 (100%)		
2. ≥3	2 (3.4%)	2 (3.3%)			1 (3.1%)	2 (6.7%)			1 (3.7%)	0		
Smoking			1.00	1.00			0.589	1.00			0.410	1.00
Yes	30 (50.8%)	30 (50.0%)			23 (71.9%)	19 (63.3%)			7 (25.9%)	11 (36.7%)		
No	29 (49.2%)	30 (50.0%)			9 (28.1%)	11 (36.7%)			20 (74.1%)	19 (63.3%)		
Alcohol abuse			1.00	1.00			0.607	1.00				
Yes	1 (1.7%)	2 (3.3%)			1 (3.1%)	2 (6.7%)			0	0		
No	58 (98.3%)	58 (96.7%)			31 (96.9%)	28 (93.3%)			27 (100%)	30 (100%)		

RT, radiotherapy; CRT, chemoradiotherapy, BMI, body mass index; NRS 2002, Nutritional Risk Score.

Table S5. Overall survival (OS) in patients: univariate and multivariate analysis for pre-treatment parameters.

Variable	Univariate analysis			Multivariate analysis	
	n	HR (95%CI)	**p-value**	HR (95%CI)	**p-value**
CRP<br>>3.50 vs. 3.50	120	2.50<br>(1.28-4.88)	**0.007**	2.22<br>1.12-4.42	**0.023**
Albumin [g/l]<br>>40.0 vs. <40.0	119	1.41<br>(0.70-2.83)	0.337		
Hb 0 [g/dl]<br>>13.5 vs. <13.5	127	0.82<br>(0.43-1.55)	0.544		
TLC 0 [/mm3]<br>>1.28 vs. <1.28	127	0.62<br>(0.30-1.28)	0.196		
PNI >39.01 vs. <39.01	119	1.08<br>(0.50-2.30)	0.845		
Age >60 vs. ≤60	127	0.86<br>(0.46-1.63)	0.653		
Gender F vs. M	127	0.49<br>(0.22-1.07)	**0.073**	0.32<br>0.13-0.82	**0.017**
General location<br>tonsil vs. others	127	1.60<br>(0.73-3.49)	0.239	3.20<br>1.19-8.60	**0.021**
Tumor depth (T)<br>T3-4 vs. T1-2	126	1.02<br>(0.53-1.94)	0. 962		
Lymph node metastasis<br>N 2-3 vs. N 0-1	126	1.09<br>(0.57-2.09)	0.791		
Radiotherapy vs.<br>Radiochemotherapy	127	1.28<br>(0.63-2.58)	0.493		

BMI 0<br>>24.53 vs. <24.53	127	0.37<br>(0.19-0.70)	**0.003**	0.32<br>0.14-0.72	**0.006**
NRS 2002<br>>1 vs. ≤1	126	3.99<br>(1.66-9.62)	**0.002**		
Smoking<br>Yes vs. No	127	1.40<br>(0.74-2.67)	0.306	0.48<br>0.22-1.07	**0.074**
Alcohol abuse<br>Yes vs. No	127	6.38<br>(1.89-21.51)	**0.003**	3.86<br>1.00-14.86	**0.050**
HPV+ vs. HPV-	127	0.31<br>(0.16-0.63)	**0.001**	0.36<br>0.16-0.80	**0.012**

0, before treatment; Hb, haemoglobin level; TLC, lymphocyte count .

Table S6. Disease-free survival (DFS) in patients: univariate and multivariate analysis for pre-treatment parameters.

Variable	Univariate analysis			Multivariate analysis	
	n	HR<br>(95%CI)	**p-value**	HR<br>(95%CI)	**p-value**
CRP<br>>3.50 vs. 3.50	120	4.00<br>1.87-8.56	**0.0004**	3.53<br>1.50-8.32	**0.004**
Albumin [g/l]<br>>40.0 vs. <40.0	119	0.86<br>0.42-1.77	0.677		
Hb 0 [g/dl]<br>>13.5 vs. <13.5	127	0.50<br>(0.25-1.00)	**0.051**		
TLC 0 [/mm3]<br>>1.28 vs. <1.28	127	0.44<br>(0.21-0.92)	**0.028**		
PNI >39.01 vs. <39.01	119	0.48<br>0.23-0.99	**0.046**	0.47<br>0.21-1.06	**0.070**
Age >60 vs. ≤60	127	0.83<br>0.42-1.65	0.604		

Gender F vs. M	127	0.86<br>0.41-1.81	0.693		
General location<br>tonsil vs. others	127	1.33<br>0.60-2.94	0.489	2.74<br>0.97-7.70	**0.056**
Tumor depth (T)<br>T3-4 vs. T1-2	126	1.22<br>0.61-2.45	0.578		
Lymph node metastasis<br>N 2-3 vs. N 0-1	126	2.11<br>(0.98-4.54)	**0.057**	3.28<br>(1.33-8.12)	**0.010**
Radiotherapy vs.<br>Radiochemotherapy	127	0.93<br>(0.42-2.06)	0.854		
BMI 0<br>>24.53 vs. <24.53	127	0.32<br>0.16-0.64	**0.001**		
NRS 2002<br>>1 vs. ≤1	126	5.05<br>2.07-12.34	**0.0004**	5.92<br>1.84-19.05	**0.003**
Smoking<br>Yes vs. No	127	1.39<br>0.70-2.78	0.346		
Alcohol abuse<br>Yes vs. No	127	4.35<br>1.03-18.35	**0.046**	5.78<br>1.04-31.97	**0.045**
HPV+ vs. HPV-	127	0.28<br>(0.12-0.62)	**0.002**	0.33<br>0.14-0.81	**0.015**

0, before treatment; Hb, haemoglobin level; TLC, lymphocyte count .

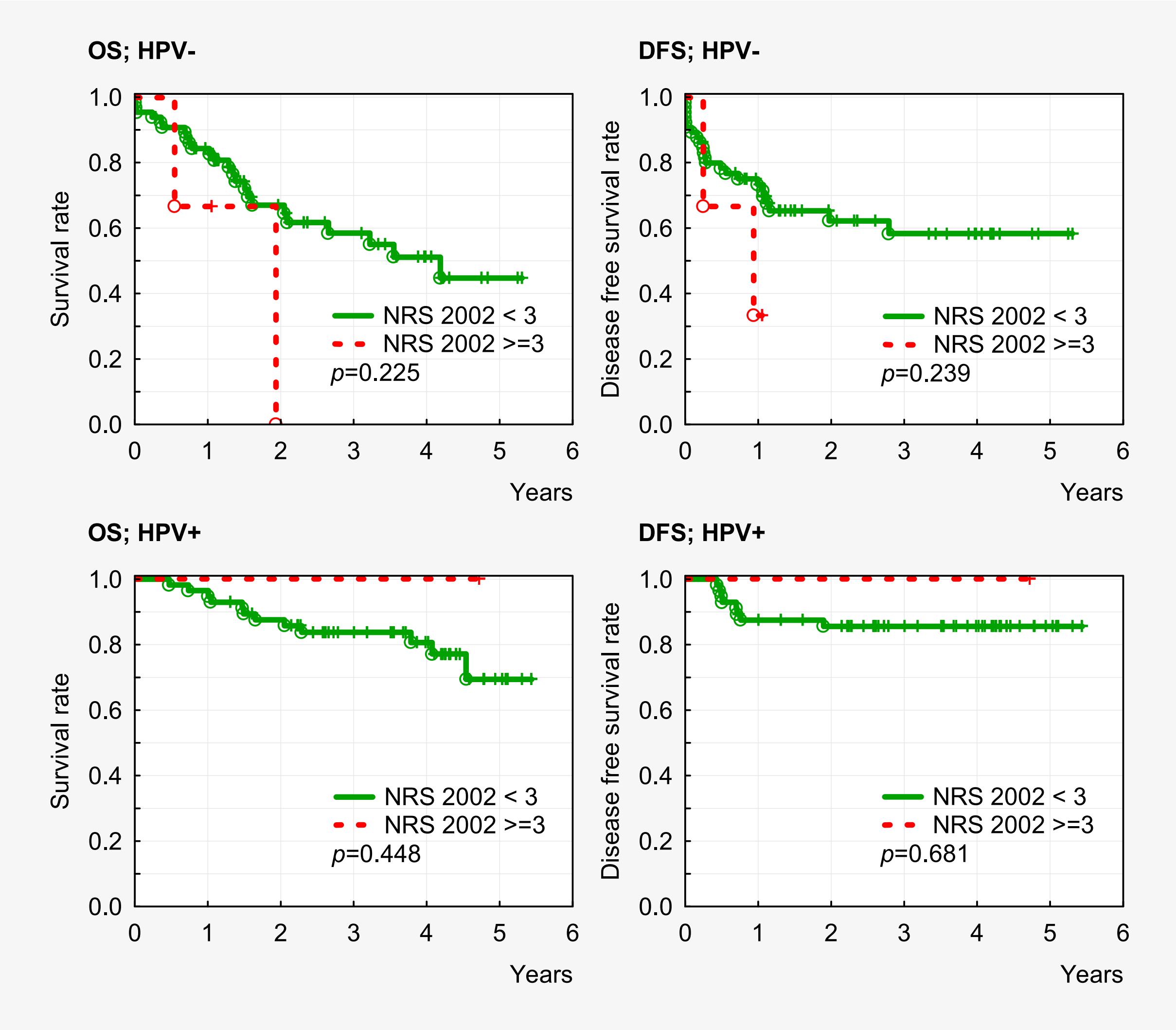

Figure S1. Overall and disease-free survival in HPV- and HPV+ patients depending on NRS 2002 (NRS 2002<3 vs. NRS 2002≥3).

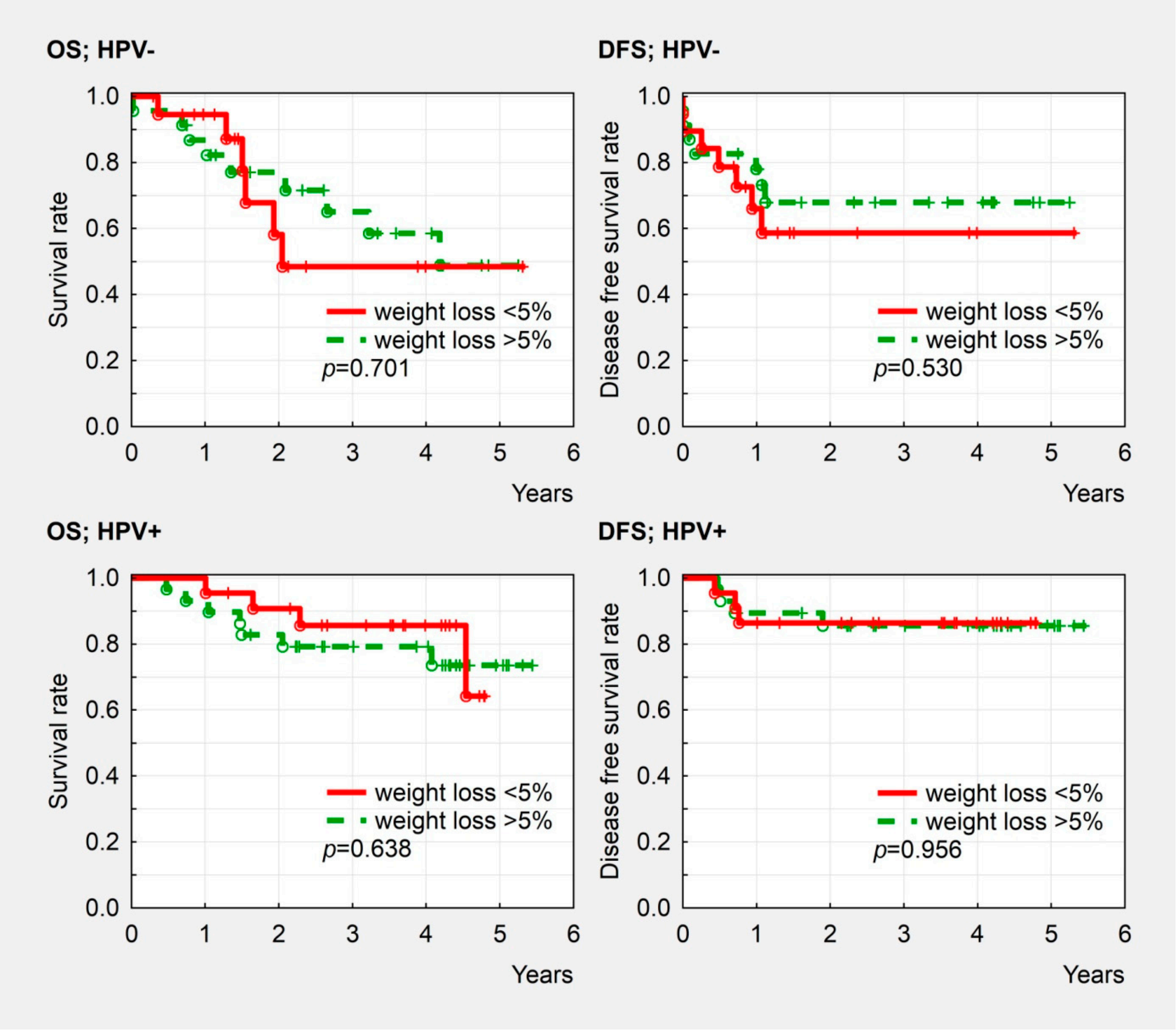

Figure S2. Overall and disease-free survival in HPV- and HPV+ patients depending on NRS 2002 (weight loss<5% vs. weight loss>5%).
